# Personality moderates the links of social identity with work motivation and job searching

**DOI:** 10.3389/fpsyg.2014.01044

**Published:** 2014-09-17

**Authors:** Pieter E. Baay, Marcel A. G. van Aken, Tanja van der Lippe, Denise T. D. de Ridder

**Affiliations:** ^1^Developmental Psychology, Utrecht UniversityUtrecht, Netherlands; ^2^Sociology, Utrecht UniversityUtrecht, Netherlands; ^3^Clinical and Health Psychology, Utrecht UniversityUtrecht, Netherlands

**Keywords:** personality, social identity, group norms, work motivation, job search behavior, school-to-work transitions

## Abstract

Work motivation is critical for successful school-to-work transitions, but little is known about its determinants among labor market entrants. Applying a social identity framework, we examined whether work motivation and job searching are social-contextually determined. We expected that some job seekers are more sensitive to contextual influence, depending on their personality. Mediation analyses on 591 Dutch vocational training students indicate that the perception of more positive work norms in someone's social context was related to higher levels of intrinsic motivation, which in turn predicted higher preparatory job search behavior and job search intentions. Multi-group analysis shows that perceived work norms more strongly predict work motivation among overcontrollers compared to resilients and undercontrollers. In conclusion, work motivation and job searching appear contextually determined: especially among those sensitive to contextual influence, people seem to work when they believe that is what people like them do.

## Introduction

Motivation and success go together well. Focusing on the domain of work, abundant research has shown that motivated employees are more involved and perform better in their job (e.g., van Knippenberg and Schie, 2000; Barrick et al., 2002), while motivated labor market entrants are more involved and successful in their job search process (see Kanfer et al., 2001, for a meta-analysis). Despite the importance of work motivation, its determinants have solely been studied among employees. Shedding light on determinants of work motivation among labor market entrants may be especially relevant given their comparatively precarious labor market situation (U.S. Department of Labor, Bureau of Labor Statistics, 2014; Statistics Netherlands, 2014). Building on the social identity framework that has been used among employees, we examine whether labor market entrants' work motivation is also social-contextually determined. Drawing on personality research, this social-contextual influence on the job search process may be especially strong for people who are sensitive to social contexts. Therefore, we study the extent to which perceived group norms in someone's social context are relevant to individual work motivation and job search behavior, and whether these relations differ between personality types (i.e., resilients, overcontrollers, undercontrollers).

A dominant perspective on the determinants of work motivation among employees is to examine intra-individual psychological processes while accounting for social-contextual factors (Haslam, 2004; Latham and Pinder, 2005). One theory that highlights the contextual aspect of motivation is social identity theory, which contends that people act in accordance with norms of relevant social groups (Tajfel, 1978; Tajfel and Turner, 1979). Social identity theory was supported in studies observing a relation between employees' identification with their work organization and higher levels of loyalty (Tyler, 1999), job involvement (van Knippenberg and Schie, 2000), and conformity to work norms (Obschonka et al., 2012).

The social identity framework may also be relevant to the study of labor market entrants. Even though labor market entrants do not yet identify with organizational groups (e.g., department X, organization Y), identification with social groups (e.g., ethnic group X, social class Y) may also affect work norms and motivation. In the current study, we consider work norm differences from an ethnic group's point of view, because ethnic identity is salient in the period of labor market entrance (French et al., 2006). Moreover, work norms may differ between ethnic groups as a result of integrated stereotypes that generally favor the majority group with regard to positive behaviors (Allport, 1954; Oyserman et al., 2007). Finally, ethnic norm differences are potentially relevant to address ethnic minorities' difficulties in their school-to-work transition (U.S. Department of Labor, Bureau of Labor Statistics, 2014). Hence, ethnic group's work norms may be a relevant contextual determinant of work motivation and job search behavior among labor market entrants.

Social identity theory predicts that perceived norms in relevant social contexts, such as the ethnic group, influence individual beliefs and behavior (Tajfel, 1978; Tajfel and Turner, 1979). The perception of belonging to a certain ethnic group affects how people define themselves and how they behave, as they are motivated to exert effort in line with the norms of relevant social groups (van Knippenberg, 2000). For example, if an ethnic group is perceived to have a more positive work norm, individuals identifying with that group are expected to be more motivated and active in their job searching. Hence, we predict a positive relation between perceived ethnic work norms on the one hand and work motivation and job searching on the other hand.

We also predict that the motivation to work explains the relation between group norms and job searching. To examine the mediating role of work motivation, we use the most commonly used distinction in motivation research (Ryan and Deci, 2000): we differentiate between intrinsic motivation (e.g., it is inherently interesting or fun) and extrinsic motivation (e.g., it leads to money or peer acceptance). As groups and perceived norms are internalized in the self-concept, it is likely that intrinsic work motivation increases with more favorable group norms. On the other hand, perceived group norms prescribe what ought to be, which is an external reason for performing behavior, so also extrinsic motivation might increase (cf. Haslam, 2004).

In sum, we hypothesize that the positive relation between perceived ethnic group's work norms and job searching is mediated by higher (intrinsic and extrinsic) work motivation.

### Contextual sensitivity: personality prototypes

Individuals may differ greatly in their sensitivity to contextual influences. While some are compliant when they encounter contextual influences like peer pressure, others remain more autonomous in their decision-making (Steca et al., 2007; Yu et al., 2013). Previous studies have used personality as an indicator for differential sensitivity, because it is known to reflect differences in responsiveness to the environment (Denissen and Penke, 2008). One oft-used personality typology, which differentiates between overcontrollers, undercontrollers, and resilients, originates in the personality theory of ego-control and ego-resiliency by Block and Block (1980). Ego-control refers to the tendency to inhibit rather than express emotional and motivational impulses, while ego-resiliency concerns the ability to respond flexibly rather than rigidly to changing demands (Block and Block, 1980). Resilients are characterized with a high level of ego-resiliency and a medium level of ego-control. Overcontrollers and undercontrollers have similarly low levels of ego-resiliency, but differ in their ego-control (with overcontrollers having high and undercontrollers having low levels) (Asendorpf and van Aken, 1999).

Studies that use the personality typology to examine individual differences in contextual sensitivity consistently find resilients to be different from the other types. Some studies have distinguished between resilients and non-resilients (i.e., combining overcontrollers and undercontrollers into one group) and they find that resilients are less strongly influenced by their environment (O'Connor and Dvorak, 2001; Nieuwenhuis et al., 2013). Possible underlying mechanisms are resilients' ability to cope flexibly with their environment (Hart et al., 2005) and to remain autonomous in their decision-making (Allen et al., 2006). Studies that additionally differentiate between overcontrollers and undercontrollers suggest that both groups may be sensitive to different aspects of their context (Dubas et al., 2002; van Aken and Dubas, 2004). When focusing on contextual effects like group norms, especially overcontrollers are sensitive to their environment. For example, overcontrollers report lower self-efficacy in resisting peers' pressure to act in line with the group (Steca et al., 2007), which is corroborated by the finding that especially delinquency of overcontrollers is influenced by the delinquency of their friends (Yu et al., 2013). Overcontrollers' vulnerability to contextual norms could be attributed to their relatively low levels of decisiveness and independence (Caspi and Silva, 1995; Hart et al., 1997). Because the current study focuses on group norms as contextual effect, we predict that overcontrollers will be especially sensitive to these norms.

Specifically, we hypothesize that the relation between ethnic group's work norms and work motivation, as well as the mediating role of work motivation in the relation between work norms and job search behavior, is stronger among overcontrollers than among resilients and undercontrollers.

## Materials and methods

### Sample and procedures

Data were collected as part of the larger longitudinal study “School2Work” on the school-to-work transition of vocational training students in the Netherlands. A cohort of students is followed from their final year of education until 3 years later (see Baay et al., 2014, for an extensive description of the project and data collection process). The current study uses the first wave of data, during which students were in their final year of vocational education.

1766 prospective vocational graduates participated in the first wave. For the present study, 750 students who intended to work upon graduation were eligible. Hence, we excluded those who intended to continue their education (*n* = 974)^1^, intended to do something else after graduation (e.g., go traveling; *n* = 28), did not know what to do after graduation (*n* = 9) or who did not indicate their plan (*n* = 5). Participants were excluded if they planned not to graduate by the end of the academic year (*n* = 62), if the questionnaire was not filled out completely (*n* = 10) or seriously (e.g., answering a series of 30 personality items with “neutral”) (*n* = 42). Given the study purposes, participants were also excluded if they identified with multiple ethnic groups (*n* = 33) or with no ethnic group at all (*n* = 12). These exclusions resulted in a final sample of 591 vocational training graduates who expected to complete their education within 6 months and whose plan was to work afterwards ^2^.

The mean age of the resulting sample was 21.49 years (*SD* = 4.61); 56% of the respondents were female; 32% was a first or second generation immigrant, having at least one parent who was born abroad. The four largest ethnic minority groups in the sample had their roots in Morocco (9.5%), Turkey (5.8%), Suriname (2.4%), and the Netherlands Antilles (1.4%).

Questionnaires were filled out in class under supervision of the students' career counselor and a research assistant. In line with school regulations, research assistants made an appointment with classes through career counselors, introduced the project in class and asked students to participate. Participation was voluntary. Students who filled out their e-mail address participated in a raffle of 12 vouchers of 25 Euros.

### Measures

Questionnaires were collected in Dutch. For existing scales that had not been used in previous Dutch studies, one researcher translated the English items into Dutch, after which two other researchers provided feedback on the translation. For both newly translated and previously used scales, the educational level of the vocational students in the current study was taken into account. Hence, long sentences and difficult terminology was avoided as much as possible. If the researchers agreed that the items represented the original items and were comprehensible for the sample, items were tested in a pilot study. While filling out the pilot questionnaire, vocational training students were encouraged to provide feedback on the comprehensibility of the questions. If necessary, adaptations were made before the items were used in the current project.

#### Personality prototypes

We based the personality prototypes on the Big Five, which we assessed with a shortened version of Goldberg's Big Five questionnaire (Goldberg, 1992; Gerris et al., 1998). All five personality traits were measured with six items, on which participants indicated whether they agreed this was characteristic of them on a 7-point scale from 1 “completely disagree” to 7 “completely agree.” Cronbach's alphas indicate that internal consistency was satisfactory (extraversion = 0.84, conscientiousness = 0.82, agreeableness = 0.78, emotional stability = 0.78, openness to experience = 0.69). As a standard procedure when deriving personality types (e.g., Dubas et al., 2002), we z-standardized the personality trait scores and excluded (four) scores that were more than 3.5 standard deviations from the mean.^3^

Latent Class Analysis (LCA) was performed to examine the number of prototypes that could be identified in our data. To decide on the number of classes, we considered the interpretability of the classes as well as the best model fit indices for LCA: Bayesian Information Criterion (BIC) and Bootstrapped Likelihood Ratio Test (BLRT) (Nylund et al., 2007). Lower BIC values indicate that the model provides a better representation of the data, while BLRT allows to statistically test whether a model with *k* + 1 classes is superior to the more parsimonious model with *k* classes. Simulations showed that LRTs should be interpreted cautiously when class sizes are small (i.e., 5% of the data) as these LRTs may be too likely to reject the more parsimonious model (Nylund et al., 2007). A comparison of the BIC values for LCAs with two (*BIC* = 9445.28), three (*BIC* = 7917.13), and four (*BIC* = 9201.62) classes favored the model with three classes. The BLRT also shows that the model with three classes provides a better fit than the two-class model [Δχ^2^_(6)_ = 86.73, *p* < 0.001]. The model with four classes provides a better fit than the three-class model [Δχ^2^_(6)_ = 130.96, *p* < 0.001], but this may be less trustworthy due to two small class sizes (*n* = 14, 2.4% and *n* = 33, 5.6%). Based on the interpretability as well as the fit indices of the three-class solution, we used the class probabilities to assign respondents to one of three prototypes.

**Figure 1:**
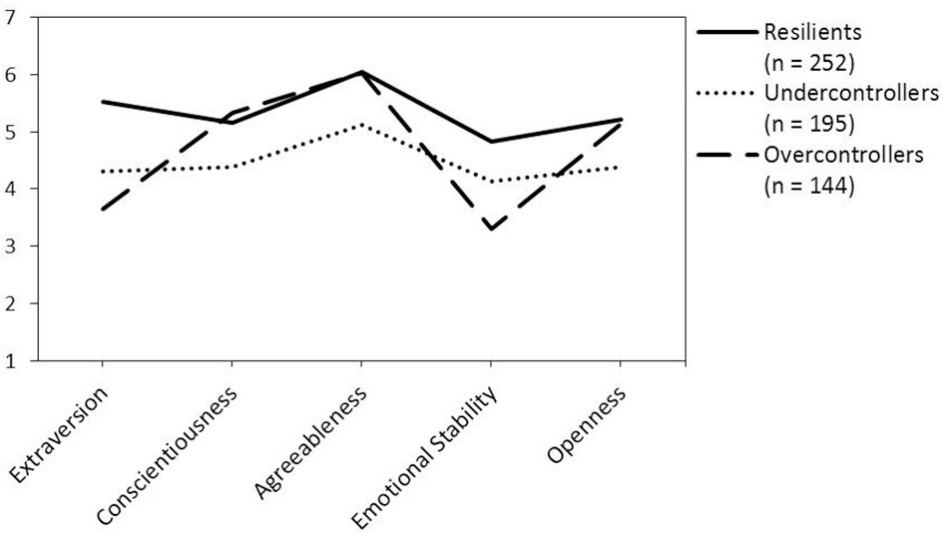
**The personality prototypes and their relations with the Big Five personality traits**.

The personality trait characteristics of the three personality prototypes align well with previous studies. Figure 1 shows that the 252 resilients in our sample had high levels on all five personality traits. The 195 undercontrollers were lower on all these traits, while the 144 overcontrollers were only lower (than both resilients and undercontrollers) on extraversion and emotional stability. This pattern is highly similar to other studies, although our overcontrollers seem more open.

#### Ethnic group's work norm

All participants rated the perceived work norm in Dutch culture on a 4-item, 7-point scale (1 = completely disagree, 7 = completely agree). Items started with “In Dutch culture…” and read (1) work is very important, (2) work determines who you are, (3) people feel very bound to work, (4) work is an important part of life. Internal consistency was satisfactory (α = 0.70). Participants who had indicated they also identified with another group besides the majority group (*n* = 179) were asked about the perceived work norm in this culture. Items were the same as for the perceived Dutch work norm, except for the beginning “In my culture….” Internal consistency was high (α = 0.82). We used the perceived norm of the ethnic group students identified most with^4^: the Dutch majority group (*n* = 457) or their minority group (*n* = 134).

The use of ethnic-specific work norms is supported in several ways. First, Exploratory Factor Analyses on the eight items measuring work norms of the Dutch and other ethnic culture favor a two-factor over a one-factor solution [χ^2^_(7, *n* = 179)_ = 165.33, *p* < 0.001], in which the two factors represent the work norms of the Dutch vs. other ethnic culture. Cross-loadings are not higher than 0.16; the correlation between the two factors is moderate (*r* = 0.32, *p* < 0.001). Second, participants who identify with the Dutch and another ethnic culture differentiate between the norms of those groups, as they report a significantly higher work norm in the Dutch culture (*M* = 5.80, *SD* = 0.91) compared to their other culture (*M* = 5.36, *SD* = 1.12), *t* = 4.89, *p* < 0.001, *d* = 0.43. Finally, among those who identify more strongly with another group than the Dutch, testing for differences in parameters of both norms in regression analyses shows that the work norm of the other ethnic culture is more predictive of work-related motivation and -behavior than the Dutch norm (Δ*b* = 0.18, *p_diff_* = 0.067 for intrinsic work motivation, Δ*b* = 0.17, *p_diff_* = 0.052 for extrinsic work motivation, Δ*b* = 0.04, *p_diff_* = 0.636 for preparatory job search behavior, Δ*b* = 0.46, *p_diff_* = 0.028 for job search intentions). In sum, participants consider work norms of the Dutch and their other ethnic group as distinct, and these norms relate differentially to their work-related motivation and -behavior.

#### Work motivation

Intrinsic motivation was measured with the identified regulation subscale of the Self-Regulation Questionnaire—Job Searching (Vansteenkiste et al., 2004). The subscale originally consists of six items but one item was dropped because two items were considered too much alike in Dutch (“I am going to work because I would like to work” and “I am going to work because I find it fun to work”; the former was dropped). The remaining five items were measured on a 7-point scale (1 = completely disagree, 7 = completely agree). Higher scores indicate higher intrinsic work motivation (α = 0.84).

Extrinsic motivation was measured with an abbreviated 5-item version of the external regulation subscale and introjected regulation subscale of the Self-Regulation Questionnaire—Job Searching (Vansteenkiste et al., 2004). The items were measured on a 7-point scale (1 = completely disagree, 7 = completely agree). One sample item reads “I am going to work because that is what one should do.” Higher scores indicate higher extrinsic work motivation (α = 0.71).

#### Job searching

Job searching was assessed with two measures. As the sample consists of prospective graduates, we included a measure of preparatory job search behavior and job search intentions. Preparatory job search behavior was assessed by an 8-item index (Blau, 1994) that measured how often participants had performed job search related activities. Sample items include “making inquiries/reading about getting a job” and “talking with people from school about possible job leads.” Respondents rated the frequency on a 5-point scale (1 = Never, 5 = More than 10 times). Internal consistency was high (α = 0.84).

Job search intentions were measured with two items: “During the upcoming months, how much effort will you put in finding a job?” (1 = No effort at all, 7 = Very much) and “how much time will you invest in job searching?” (1 = Less than once a month, 6 = Every day). Higher scores indicate higher job search intentions (*r* = 0.71). Due to uneven measurement scales, items were z-standardized before taking the mean.

## Results

Descriptive statistics and bivariate correlations of the study variables can be found in Table 1. In line with expectations, perceived work norms are positively correlated with indicators of work motivation and job searching.

**Table 1:** **Descriptive statistics and bivariate correlations of study variables**.

	***M***	***SD***	**4**	**5**	**6**	**7**	**8**	**9**	**10**	**11**	**12**	**13**
1. Resilient	0.43	0.49	0.66^***^	0.32^***^	0.18^***^	0.43^***^	0.50^***^	0.04	0.17^***^	−0.02	0.10^*^	0.02
2. Overcontroller	0.24	0.43	−0.51^***^	0.14^***^	0.22^***^	0.26^***^	−0.51^***^	0.04	0.08^†^	0.05	0.00	0.00
3. Undercontroller	0.33	0.47	−0.23^***^	−0.47^***^	−0.38^***^	−0.69^***^	−0.07	−0.08^†^	−0.25^***^	−0.02	−0.11^**^	−0.02
4. Extraversion	4.67	1.12		0.09^*^	−0.04	0.19^***^	0.48^***^	0.03	0.10^*^	0.07	0.08^†^	−0.01
5. Openness to experience	4.92	0.81			0.19^***^	0.38^***^	0.04	0.11^**^	0.24^***^	0.09^*^	0.15^***^	0.02
6. Conscientiousness	4.95	1.01				0.30^***^	−0.02	0.16^***^	0.23^***^	0.05	0.10^*^	0.15^**^
7. Agreeableness	5.72	0.66					0.03	0.11^**^	0.29^***^	0.10^*^	0.15^**^	0.07
8. Emotional stability	4.23	1.04						0.00	−0.03	−0.13^**^	−0.01	−0.05
9. Ethnic work norm	5.55	0.88							0.36^***^	0.23^***^	0.12^**^	0.11^*^
10. Intrinsic work motivation	5.70	0.90								0.39^***^	0.23^***^	0.18^***^
11. Extrinsic work motivation	4.59	1.16									0.09^*^	0.11^*^
12. Prep. job search behavior	1.62	1.09										0.31^***^
13. Job search intentions^a^^,^^b^	3.84	1.61										

*** p < 0.001;

** p < 0.01;

* p < 0.05;

† *p < 0.10*.

a *Due to uneven measurement scales, items were z-standardized before the scale was constructed. For illustrative purposes, the scale mean of the uncentered items is reported in Table 1*.

b *The measure of job search intentions was only relevant for those who had not yet found employment for after graduation. As 123 prospective graduates had already found employment, this measure was relevant to 468 respondents*.

Hypotheses were tested in a Structural Equation Modeling (SEM) framework in Mplus 7.0. The hypothesis that the relation between perceived work norms and job searching is mediated by work motivation was tested with Ordinary Least Squares regression analysis. For both direct and indirect relations, bootstrapped analyses were performed to account for potential non-normal variable distributions. In bootstrapping, random samples are generated based on the original data (in the current analyses, 1000 sets). For each random sample, the direct and mediated effects were computed. The distribution of these effects was then used to obtain bootstrapped 95% confidence intervals for the size of the effects.

The positive relation between work norms and job searching was mediated by higher levels of work motivation.^5^ First, a higher perceived work norm was related to higher levels of intrinsic motivation (*b* = 0.61, 95% CI 0.40–0.89, *t* = 5.17, *p* < 0.001) and extrinsic motivation (*b* = 0.30, 95% CI 0.19–0.50, *t* = 4.05, *p* < 0.001). Second, and portrayed in Table 2, the direct effect of the perceived work norm on preparatory job search behavior was completely mediated by work motivation. More specifically, testing for indirect effects shows intrinsic motivation to mediate the relation between perceived work norm and preparatory job search behavior (*b* = 0.10, 95% CI 0.04–0.17, *t* = 3.25, *p* = 0.001), whereas extrinsic motivation did not mediate this relation (*b* = 0.00, 95% CI −0.03–0.04, *t* = 0.15, *p* = 0.882). The same pattern was observed for job search intentions, with significant mediation of intrinsic motivation (*b* = 0.08, 95% CI 0.01–0.14, *t* = 2.36, *p* = 0.018), and no mediation of extrinsic motivation (*b* = 0.02, 95% CI −0.02–0.06, *t* = 0.86, *p* = 0.390). Indeed, a higher perceived work norm related to more job searching because of a higher (intrinsic) motivation to work.

**Table 2:** **Motivation mediates the relation between perceived own group's work norm and job searching**.

	**Preparatory job search behavior**						**Job search intentions**					
	***b***	***SE***	**95% CI**	***b***	***SE***	**95% CI**	***b***	***SE***	**95% CI**	***b***	***SE***	**95% CI**
Perceived work norm	0.23^**^	0.09	0.07–0.41	0.08	0.09	−0.09–0.26	0.45^*^	0.18	0.11–0.80	0.19	0.21	−0.24–0.59
Intrinsic work motivation				0.25^***^	0.07	0.12–0.38				0.34^*^	0.16	0.05–0.66
Extrinsic work motivation				0.01	0.08	−0.16–0.18				0.17	0.20	−0.19–0.59

*** p < 0.001;

** p < 0.01;

* *p < 0.05*.

We hypothesized that the relation between perceived group norms on the one hand and individual work motivation and job searching on the other hand would be stronger among overcontrollers than among resilients and undercontrollers. We used multiple group analysis in Mplus to analyze whether the relations between work norms, work motivation, and job searching were different between the three personality prototypes. As a baseline model, we constrained all relations to be equal for resilients, undercontrollers, and overcontrollers. In step 2, we allowed the relations between work norms and work motivation to be different for overcontrollers compared to resilients and undercontrollers. This led to a significant improvement of the model fit compared to the baseline model [Δχ^2^_(2)_ = 8.63, *p* = 0.013]. Allowing these relations to also be different for undercontrollers and resilients did not improve the model fit compared to the step-2 model [Δχ^2^_(2)_ = 0.38, *p* = 0.827]. Allowing the direct links of work norms with job searching to be different for overcontrollers compared to resilients and undercontrollers also did not improve the model fit compared to the step-2 model [Δχ^2^_(2)_ = 0.84, *p* = 0.657]^6^.

**Figure 2:**
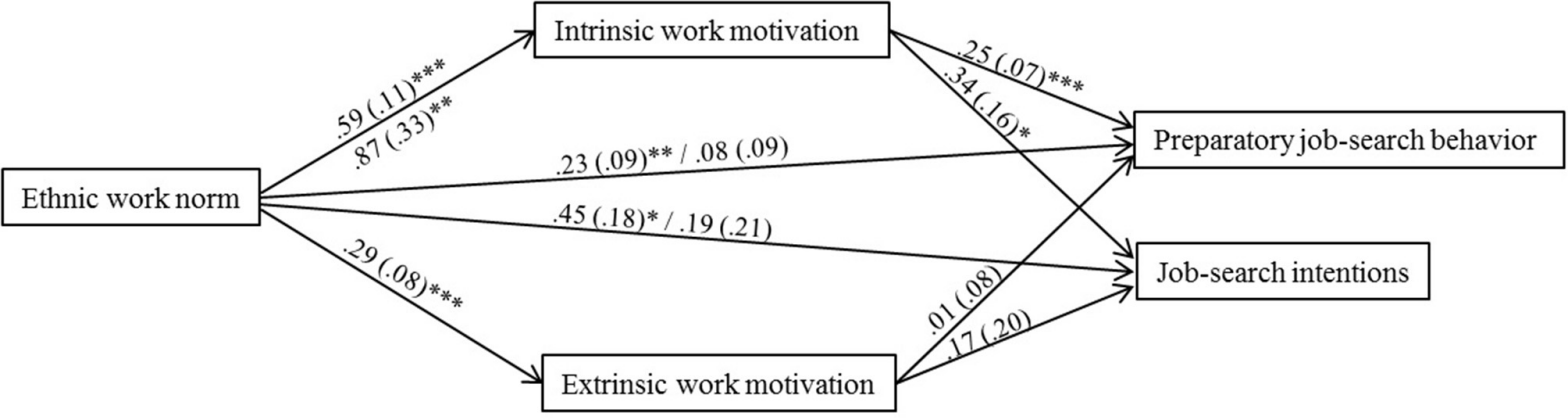
**Differential sensitivity to contextual influence among overcontrollers**. Unstandardized estimates [*b* (*SE*)] above arrows apply to undercontrollers and resilients; unstandardized estimates below arrows apply to overcontrollers. If these estimates did not significantly differ, estimates for all personality types are depicted above the arrows. Arrows from ethnic work norm to preparatory job search behavior and job search intentions depict the relations without controlling for work motivation, followed by the relations controlled for work motivation. ^***^p < 0.001; ^**^p < 0.01; ^*^p < 0.05.

Figure 2 depicts the model that best describes the data. In line with expectations, the relation between work norms and work motivation is stronger among overcontrollers compared to undercontrollers and resilients. This applies to the direct relations between work norms and intrinsic as well as extrinsic work motivation. In addition, we used moderated mediation analysis for latent interaction variables to examine whether the mediating role of work motivation is stronger among overcontrollers compared to undercontrollers and resilients. This was not the case (*b*s < 0.08, *p*s > 0.363)^7^.

## Discussion

The present study applied a social identity perspective to the concept of work motivation among labor market entrants. Although work motivation has been shown to be predictive of employment chances for labor market entrants (Kanfer et al., 2001), its social identity component had not been examined before. We found that work motivation is social-contextually determined, as work norms in job seekers' social context predicted individual work motivation and job searching. In line with expectations, sensitivity to this contextual influence was dependent on job seekers' personality.

Our results confirm the importance of perceived work norms in relevant social groups, as a higher perceived work norm in the ethnic group was related to higher levels of intrinsic motivation, which in turn predicted higher preparatory job search behavior and job search intentions. These findings align with previous organizational research that showed that identification with a group is associated with individual work-related motivation and behavior among employees (van Knippenberg and Schie, 2000; Obschonka et al., 2012). Moreover, our findings are consistent with research that measured perceived norms explicitly and found relations with motivation and behavior in the domains of health and academic achievement (Oyserman et al., 2007; Oyserman, 2008). The current study shows that perceived work norms in someone's ethnic group similarly relate to motivation and behavior in the context of job searching among labor market entrants.

Even though a higher perceived work norm was also related to higher levels of extrinsic motivation, extrinsic motivation did not predict preparatory job search behavior and job search intentions. This is consistent with a recent finding that intrinsic motivation has more favorable correlates with work related measures as compared to extrinsic motivation (Moran et al., 2012). Together, this suggests that intrinsic work motivation is more influential in work-related behavior than extrinsic work motivation.

The extent to which the social context predicted the motivation and behavior of individuals was dependent on their personality. While the relation between work norms and work motivation was significant among all personality types, it was strongest among overcontrollers. This aligns with previous research on contextual sensitivity, which had found that overcontrollers may be more sensitive to negative norms (Steca et al., 2007; Yu et al., 2013). By extending this to sensitivity of positive norms, overcontrollers seem more susceptible to contextual influence, regardless of whether these are negative or positive factors (cf. Nieuwenhuis et al., 2013).

Several features of this study strengthen the conclusions that can be drawn from our results. Theoretically, we were able to build on literature in organizational research on the role of groups in work-related behavior. While investigating work motivation among labor market entrants, we considered the role of ethnic instead of organizational groups. To acquire deeper insight into the process through which group identification affects motivation, we explicitly measured the perception of group norms, which had proven relevant in other domains of identity based motivation (Oyserman et al., 2007). Methodologically, we used a large-scale survey which is representative for Dutch vocational training graduates. With 23% of the respondents identifying most with an ethnic minority group, we were able to provide insight in majority-minority processes that can likely be generalized to the Dutch population of vocational training graduates.

Some limitations of the current study need to be addressed. First, we relied on self-report data, which means that the observed correlations may have occurred because of common method variance (Spector, 2006). Although we could not use objective measures of work norms, as social identity theory theorizes about someone's own perception of a norm, more objective measures of job searching would have improved the current study. Second, this study used cross-sectional data, which does not allow for causal inferences about the observed relations between perceived group norms and individual behavior. It seems unlikely, however, that the hypothesized direction of effects is reversed and that individual behavior influences the perceived group norm.

### Implications for research and policy

Future research could consider other social groups that influence individual motivation and behavior. The current study focused on the role of perceived ethnic group norms to facilitate comparisons with previous research on identity based motivation (e.g., Oyserman et al., 2007) and because ethnic identity is salient among late adolescents (French et al., 2006). Future research could include other social categories that have proven relevant in other domains (Oyserman and James, 2011). For example, gender roles have been related to achievement related choices (Eccles, 2011), social class norms to academic achievement (Oyserman et al., 2007), and age-related expectations to work attitudes and behavior (Rhodes, 1983). Norms in these social groups may be similarly related to work motivation and job searching among labor market entrants.

The findings of the current study might inspire interventions that aim to stimulate youth to actively search for employment. To achieve higher levels of individual motivation and job searching, as the current study shows, attention could be devoted to perceived work norms in relevant social groups. Given that work values are least stable during tertiary school (Jin and Rounds, 2012) and school peers influence each other's transitions into early adulthood (Kiuru et al., 2012), it seems advisable to especially target late adolescents. At the school level, interventions could target existing misperceptions about peer norms (Burchell et al., 2013). Previous studies have shown that adolescents overestimate peer norms, leading to norm misperceptions, in various domains (e.g., drinking, Perkins, 2007; weight, Perkins et al., 2010; drug use, McCabe, 2008). In the current study, adolescents may have similar misperceptions about work norms. To illustrate, ethnic minorities overestimated the work norm of the majority group, as they reported a more positive majority work norm than the majority group itself (results not shown). At the same time, ethnic minorities reported a lower work norm for their own group than for the majority group, which may be an underestimation of the ethnic minority's work norm. One approach to correct possible misperceptions about work norms is to ask people to estimate work norms among peers and confront them afterwards with the actual norms of these peers. When this approach was used in a study on drinking norms, the perceived norms at 3- and 6-month follow-up were more realistic and led to decreased alcohol consumption (Neighbors et al., 2004).

## Conclusion

The current study has provided new insights into the role of social groups on work motivation among labor market entrants. The importance of work in someone's ethnic group seems relevant for an individual's motivation to work and subsequent job searching. In conclusion, work motivation and job searching appear contextually determined: especially among those sensitive to contextual influence, people seem to work when they believe that is what people like them do.

### Conflict of interest statement

The authors declare that the research was conducted in the absence of any commercial or financial relationships that could be construed as a potential conflict of interest.

## Footnotes

^1^The current sample's continuation rate of 55% is representative for the continuation rate among vocational education students in the Netherlands (Statistics Netherlands, 2014).

^2^The results are robust to the exclusion rules. Compared to the analyses reported below, analyses that include participants who identify with multiple ethnic groups (*n* = 33) and analyses that include participants who plan to graduate by the following academic year (*n* = 62) give similar results with regard to the direct, indirect and moderated relations (results available upon request).

^3^Keeping the outliers in the analyses results in an extra class of two cases. The pattern for the remaining classes is very similar to the results without the outliers as reported below (results available upon request).

^4^Highest ethnic group identification was determined by comparing the extent to which participants felt Dutch (0 = not at all Dutch, 10 = very Dutch) and, if they indicated that they also identified with another group, the extent to which they identified with that group (0 = not at all, 10 = very much).

^5^The direct and indirect effects were very similar in models with and without controlling for respondents' personality type (results available upon request). For reasons of parsimony, we report the models without controlling for personality type.

^6^We took a person-centered approach and focus on personality prototypes to build on prior studies in this field and because of its parsimoniousness. A variable-centered approach would allow to explore the effects of the separate Big Five personality traits. In addition, prediction of outcomes may be greater for trait-based approaches (McCrae et al., 2006). Therefore, we have also analyzed the extent to which Big Five personality traits moderate the direct and indirect relations between work norms, work motivation, and job search behavior. The general pattern is that Big Five personality traits do not moderate these relations (results available upon request). Specifically, for the moderating role of Big Five personality traits in the relation between work norms and work motivation, only the relation between work norms and intrinsic work motivation is moderated, only by agreeableness. That is, intrinsic work motivation was less strongly related to work norms among agreeable job seekers. The mediating role of work motivation in the relation between work norms and job search behavior was generally not moderated by Big Five personality traits either. Again, the only significant effect concerned the relation between work norms and preparatory job search behavior that was mediated by intrinsic work motivation; this mediation effect was less strong among agreeable job seekers. Conscientiousness, extraversion, openness to experience, and emotional stability did not moderate the relations examined in the current paper. Correlations between the Big Five personality traits, work motivation, and job search behavior are portrayed in Table 1.

^7^The findings that the relation between norms and motivation is stronger for overcontrollers, while the relation between norms and job searching mediated by motivation is not stronger for overcontrollers may seem like a contradiction. However, these patterns can be found at the same time. That is, the significant moderation analysis shows that the motivation of overcontrollers is more strongly related to norms in their environment, while the non-significant moderated mediation analyses shows that the relation between norms and job searching is explained by (intrinsic) work motivation, regardless of personality prototype. Hence, the mechanism through which norms relate to behavior are the same for all groups (i.e., through work motivation), while some groups may be more sensitive to those norms, which is why these norms more strongly relate to their motivation.
