# Supplementary material for: Personality moderates the links of social identity with work motivation and job searching
Source: Front Psychol. 2014 Sep 17;5:1044. doi: 10.3389/fpsyg.2014.01044 (PMC4166077; doi:10.3389/fpsyg.2014.01044)
Supplement: Supplementary file 1 [file DataSheet1.DOCX]

*Supplementary Materials: Overview of translated items.*

**Perceived Dutch work norms**

*Hoe belangrijk is werk in de Nederlandse cultuur denk jij?*

In de Nederlandse cultuur..

- ... is werken heel belangrijk.

- ... bepaalt je werk wie je bent.

- … voelen mensen zich erg verbonden met hun werk.

- … is werk een belangrijk deel van het leven.

**Perceived ethnic group’s work norms**

*Je hebt aangegeven dat je je ook bij een andere groep voelt horen. De volgende vragen gaan over deze andere groep.*

In mijn cultuur:

- ... is werken heel belangrijk.

- ... bepaalt je werk wie je bent.

- … voelen mensen zich erg verbonden met hun werk.

- … is werk een belangrijk deel van het leven.

**Extrinsic work motivation**

*Waarom ga je werken?*

Ik ga werken omdat…

- … ik geld nodig heb om rond te komen

- … ik dan geld ga verdienen waarmee ik veel kan kopen

- … werken mijn plicht is in deze samenleving

- ... dat zo hoort

- … ik niet wil dat anderen denken dat ik lui ben

**Intrinsic work motivation**

*Waarom ga je werken?*

Ik ga werken omdat…

- … ik mijn talenten verder kan ontwikkelen door te werken

- … ik mijn persoonlijkheid verder kan ontwikkelen door te werken

- … werk zinvol is voor mezelf

- … ik het leuk vind om te werken

- … ik werk belangrijk vind

**Preparatory job search behavior**

Hoe vaak heb je in de afgelopen 6 maanden…

- Informatie gezocht over hoe je het beste kunt solliciteren

- Jouw eigen wensen en talenten opgeschreven om je voor te bereiden op het solliciteren

- Gepraat met familie, vrienden over het zoeken naar een baan

- Gepraat met mensen van school (buiten de lessen) over het zoeken naar een baan

- Gepraat met vroegere werkgevers/ stagecontacten over een baan voor jou

- Gepraat met iemand van een uitzendbureau, het UWV of een andere bemiddelaar over het zoeken naar een baan

- Vacatures op internet gezocht en gelezen

- Vacatures in kranten en vakbladen (niet op internet) gezocht en gelezen

**Job search intentions**

Hoeveel moeite ga je doen om een baan te vinden in de komende maanden?

Hoeveel tijd ga je besteden aan het zoeken van een baan?
